# Supplementary material for: Novel Cocrystals of Vonoprazan: Machine Learning-Assisted Discovery
Source: Pharmaceutics. 2022 Feb 16;14(2):429. doi: 10.3390/pharmaceutics14020429 (PMC8877905; doi:10.3390/pharmaceutics14020429)
Supplement: Supplementary file 1 [file pharmaceutics-14-00429-s001.zip › pharmaceutics-1576531-supplementary.pdf]

Table S1. Classification results of 51 VPZ-coformer pairs using the ANN model.

Coformer	Predicted label
Pyrocatechol (CAT)	1
Hydroquinone (HYQ)	1
Resorcinol (RES)	1
Pyrogallol (GAL)	1
Methyl-hydroquinone (MHQ)	1
Acetamide (ACT)	1
Glycolamide (GLY)	1
Benzamide (BZM)	1
Nicotinamide (NCT)	1
Isonicotinamide (INCT)	1
Urea	1
Benzoic acid (BZA)	1
Succinic acid (SUC)	1
pyroglutamic acid (PGA)	1
4-hydroxybenzoic acid (4HBA)	1
Pyrrole-2-carboxylic acid (PCA)	1
oxamic acid (OXM)	1
Saccharin (SAC)	1
Piperazine (PIP)	1
Orcinol	0
Phloroglucinol	0
L-prolinamide	0
Adipic acid	0
Suberic acid	0
Glutaric acid	0
Phthalic acid	0
4-phenylbutyric acid	0
Pimelic acid	0
cinnamic acid	0
4-aminosalicylic acid	0
6-hydroxy-2-naphthoic acid	0
Indoleacetic acid	0
Caffeic acid	0
Vanillic acid	0

3,4-dihydroxybenzoic acid	0
Trans-ferulic acid	0
Trans-p-coumaric acid	0
Aminobenzoic acid	0
Gallic acid	0
orotic acid	0
L-Asparagine	0
L-Histidine	0
L-Valine	0
L-DOPA	0
tryptophan	0
Tyrosine	0
Caffein	0
Riboflavin	0
Ascorbic acid	0
4,4’-dipyridyl (BPY)	0
1,2-Di(pyridin-4-yl)ethene	0

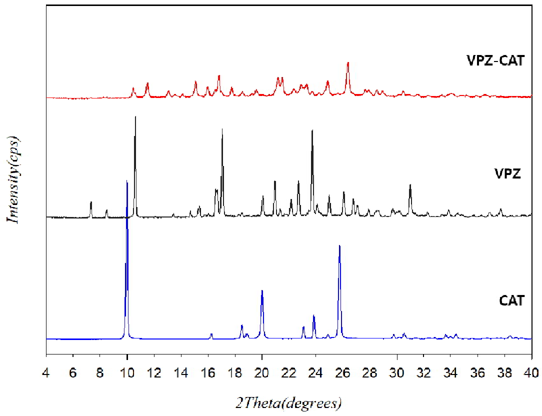

Figure S1. PXRD patterns of VPZ, CAT, and VPZ-CAT prepared by LAG.

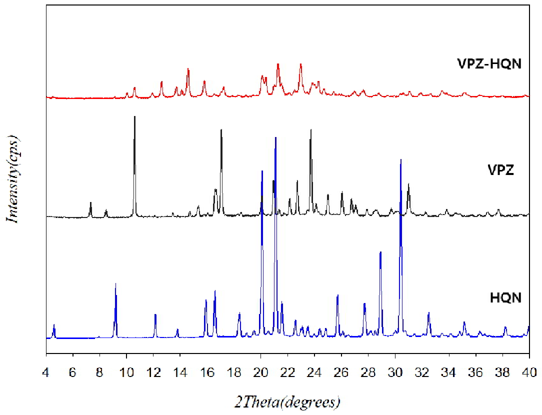

Figure S2. PXRD patterns of VPZ, HYQ, and VPZ-HYQ prepared by LAG.

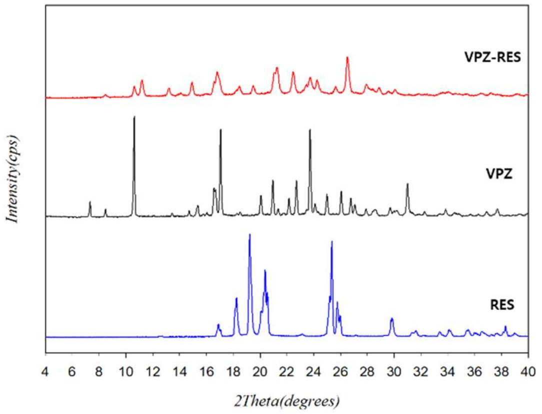

Figure S3. PXRD patterns of VPZ, RES, and VPZ-RES prepared by LAG.

.

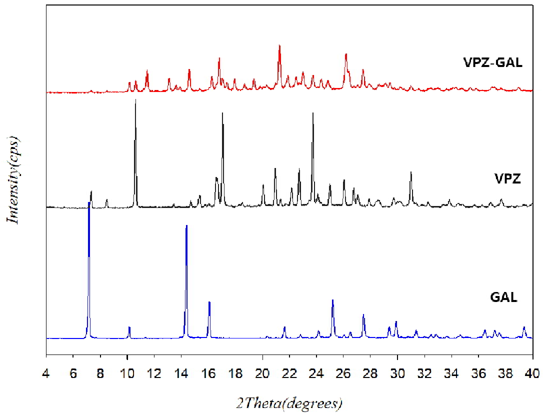

Figure S4. PXRD patterns of VPZ, GAL, and VPZ-GAL prepared by LAG.

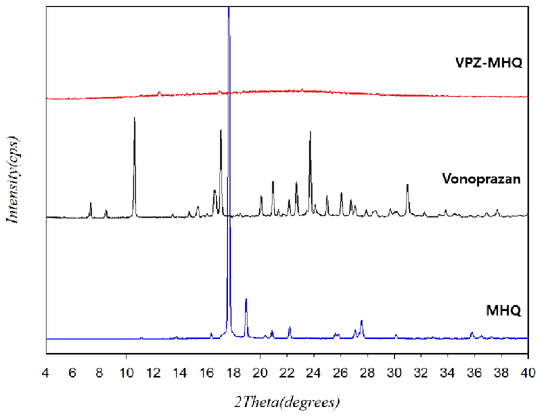

Figure S5. PXRD patterns of VPZ, MHQ, and VPZ-MHQ prepared by LAG.

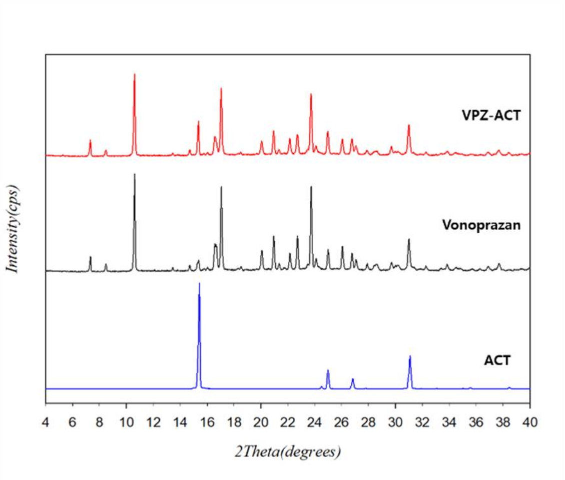

Figure S6. PXRD patterns of VPZ, ACT, and VPZ-ACT prepared by LAG.

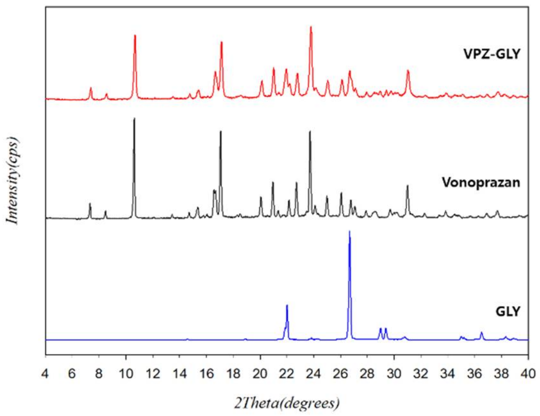

Figure S7. PXRD patterns of VPZ, GLY, and VPZ-GLY prepared by LAG.

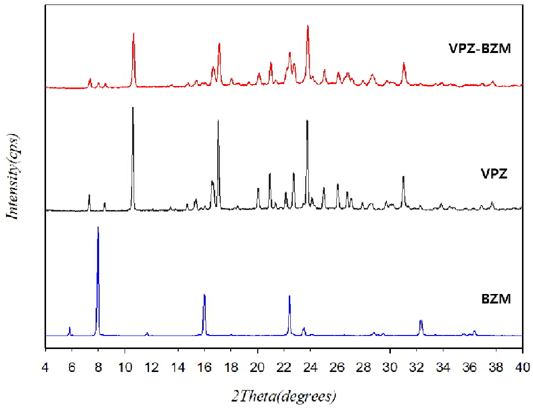

Figure S8. PXRD patterns of VPZ, BZM, and VPZ-BZM prepared by LAG.

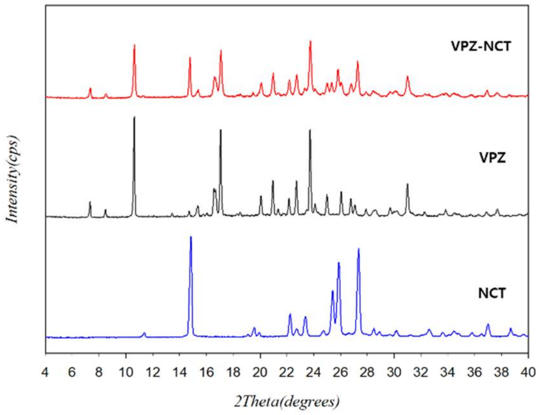

Figure S9. PXRD patterns of VPZ, NCT, and VPZ-NCT prepared by LAG.

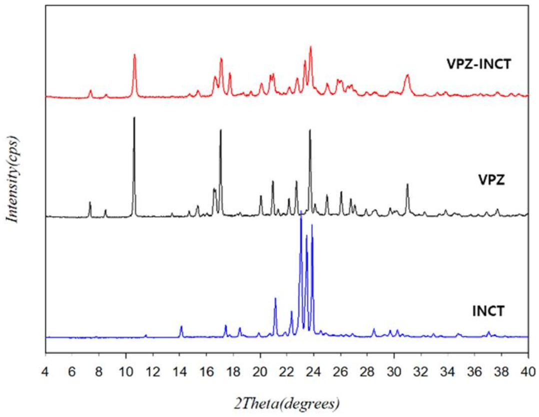

Figure S10. PXRD patterns of VPZ, INCT, and VPZ-INCT prepared by LAG.

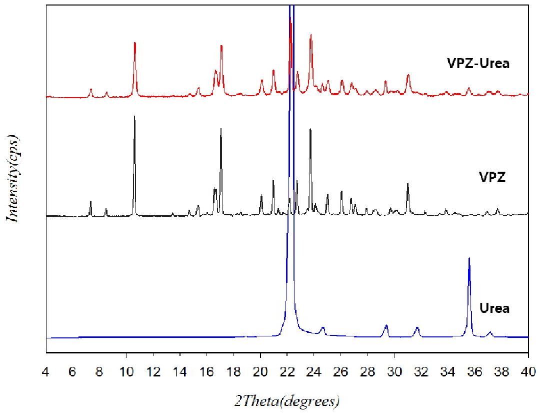

Figure S11. PXRD patterns of VPZ, Urea, and VPZ-Urea prepared by LAG.

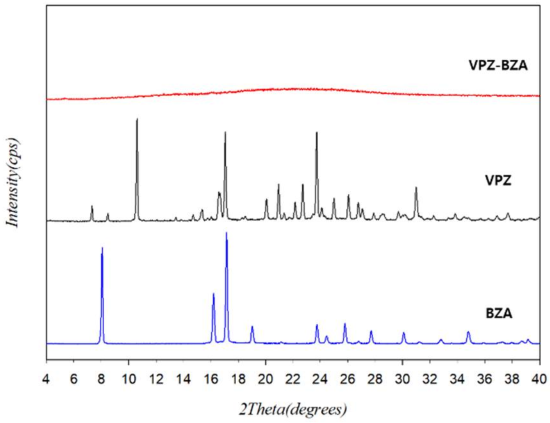

Figure S12. PXRD patterns of VPZ, BZA, and VPZ-BZA prepared by LAG.

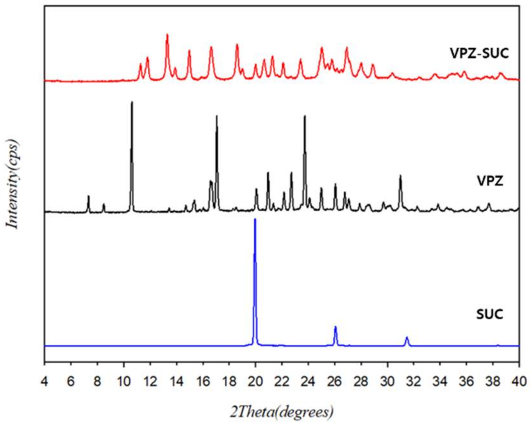

Figure S13. PXRD patterns of VPZ, SUC, and VPZ-SUC prepared by LAG.

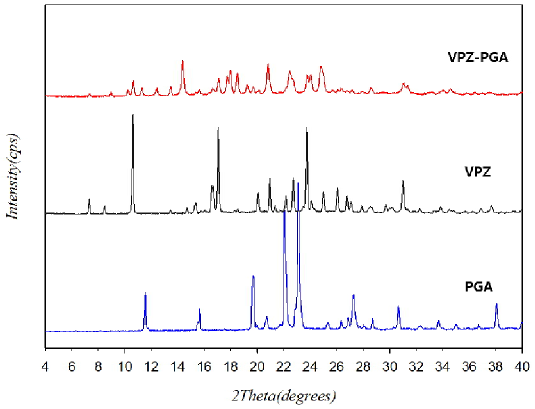

Figure S14. PXRD patterns of VPZ, PGA, and VPZ-PGA prepared by LAG.

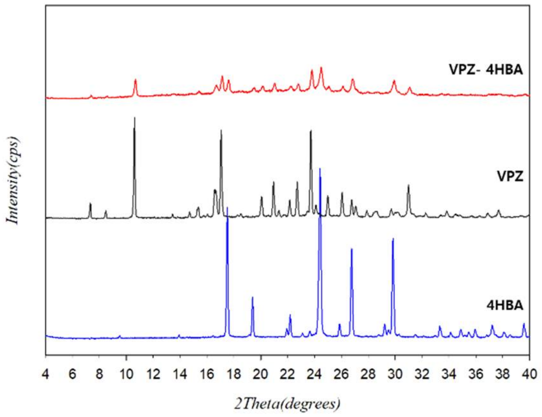

Figure S15. PXRD patterns of VPZ, 4HBA, and VPZ-4HBA prepared by LAG.

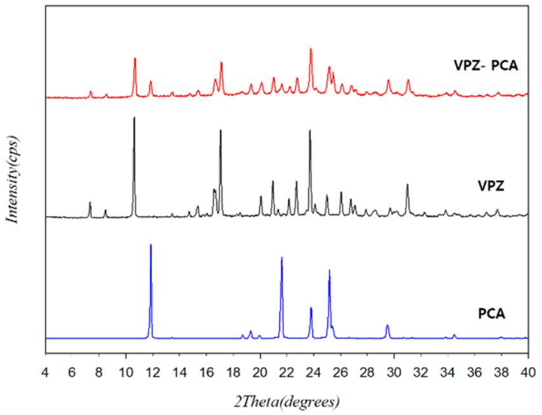

Figure S16. PXRD patterns of VPZ, PCA, and VPZ-PCA prepared by LAG.

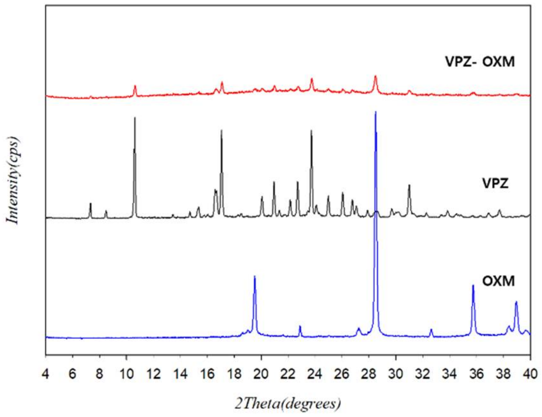

Figure S17. PXRD patterns of VPZ, OXM, and VPZ-OXM prepared by LAG.

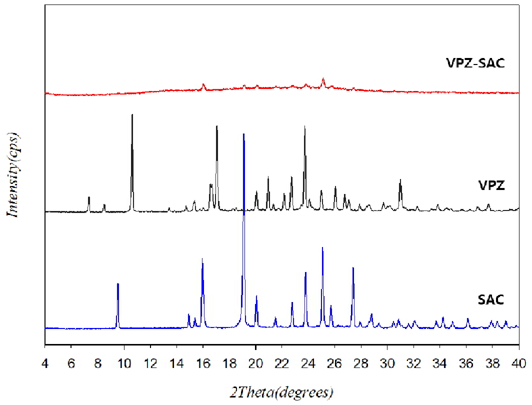

Figure S18. PXRD patterns of VPZ, SAC, and VPZ-SAC prepared by LAG.

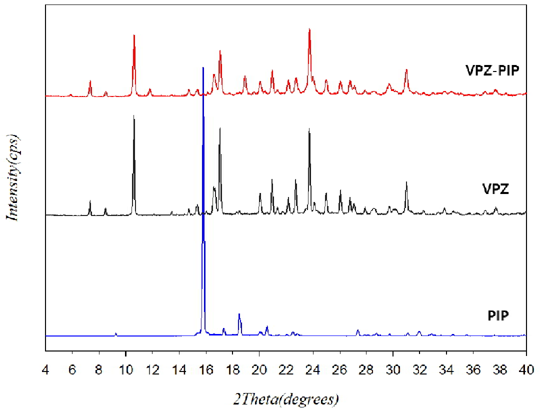

Figure S19. PXRD patterns of VPZ, PIP, and VPZ-PIP prepared by LAG.

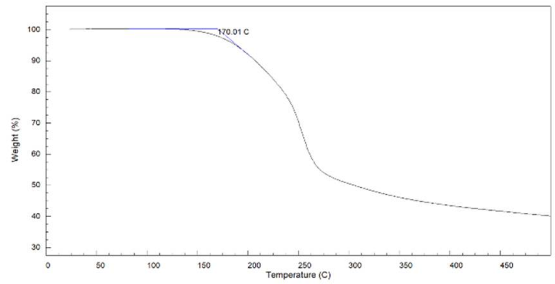

Figure S20. TGA thermogram of VPZ-CAT prepared by RC.

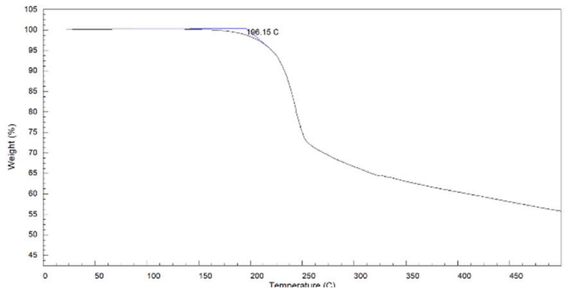

Figure S21. TGA thermogram of VPZ-GAL prepared by RC.

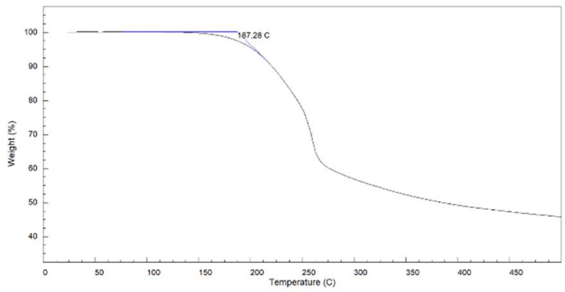

Figure S22. TGA thermogram of VPZ-RES prepared by RC.

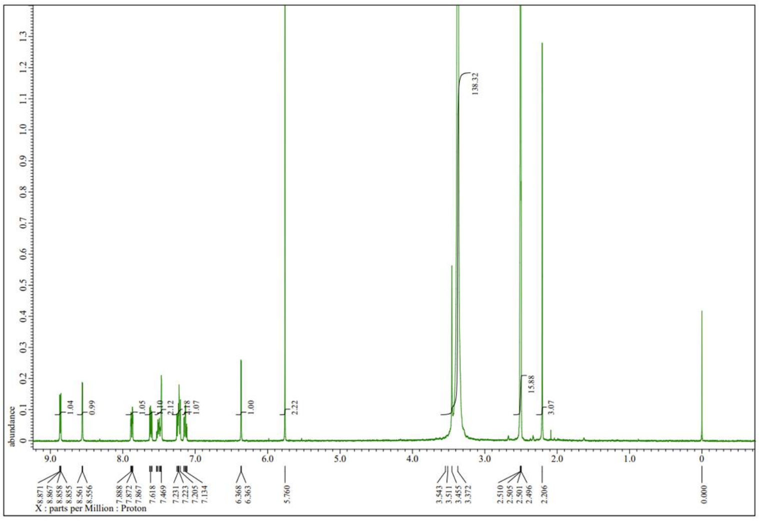

Figure S23. ^{1}H NMR of VPZ.

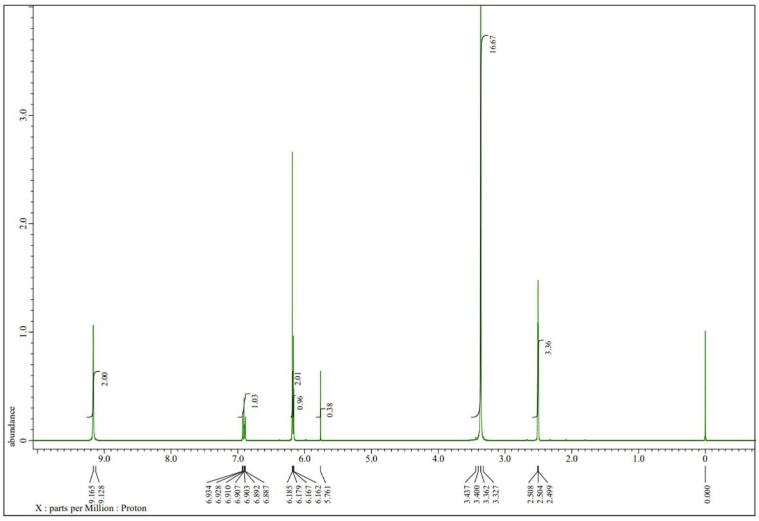

Figure S24. ^{1}H NMR of RES.

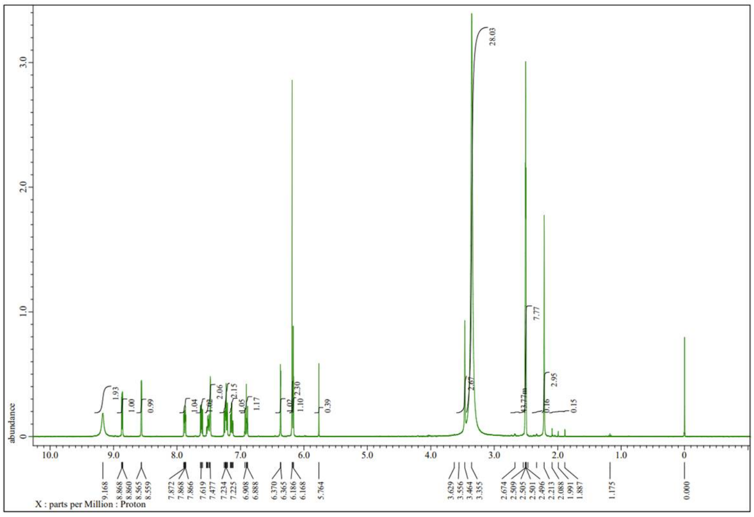

Figure S25. ^{1}H NMR of VPZ-RES.

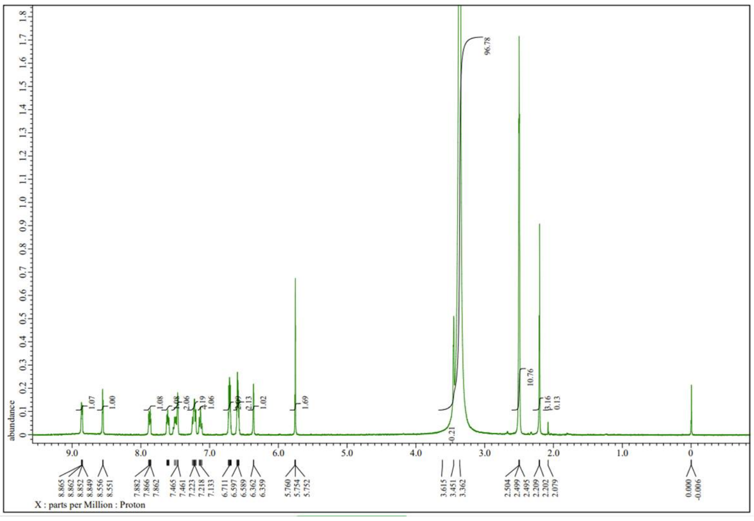

Figure S26. ^{1}H NMR of CAT.

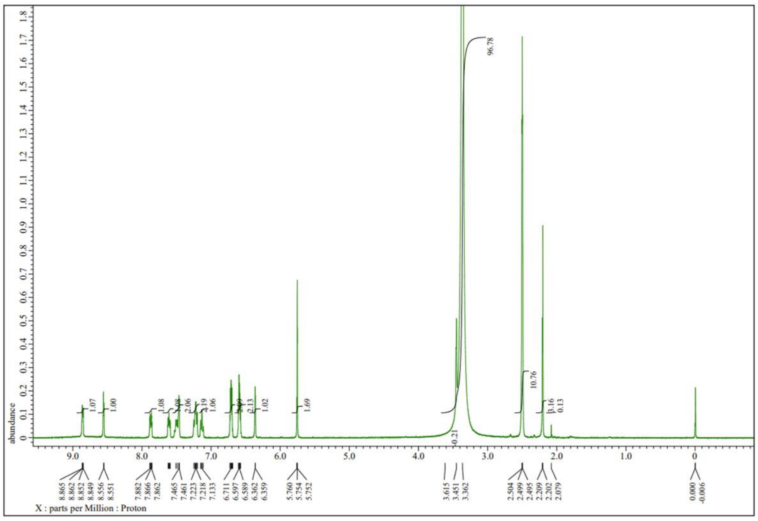

Figure S27. ^{1}H NMR of VPZ-CAT.

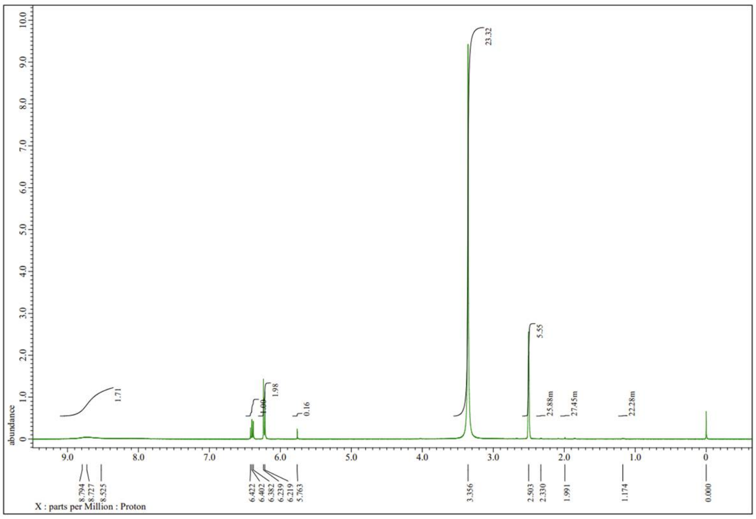

Figure S28. ^{1}H NMR of GAL.

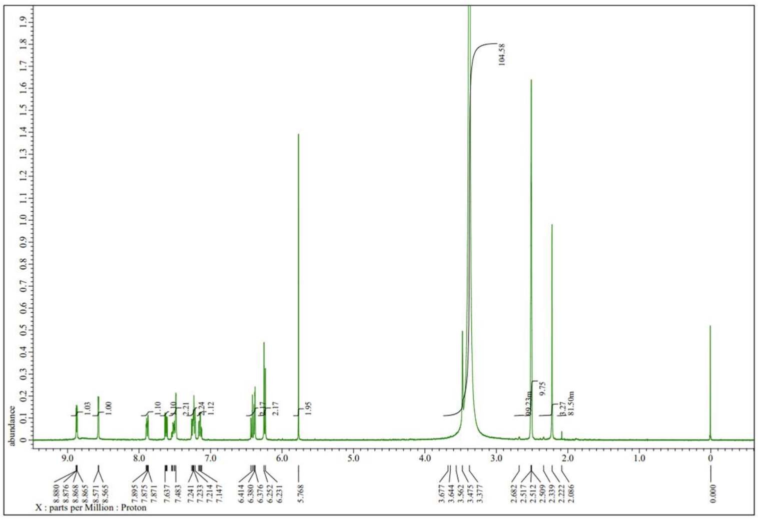

Figure S29. ^{1}H NMR of VPZ-GAL.

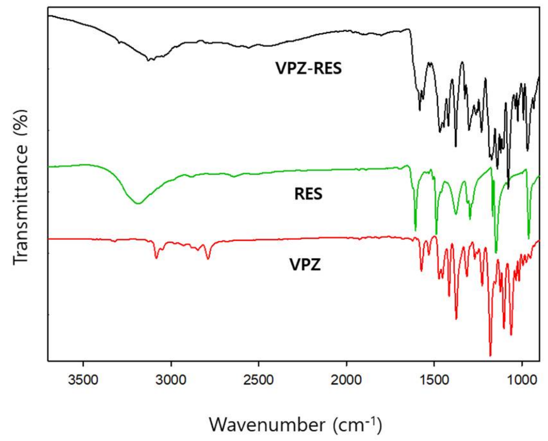

Figure S30. FT-IR spectra of VPZ, RES, and VPZ-RES prepared by RC.

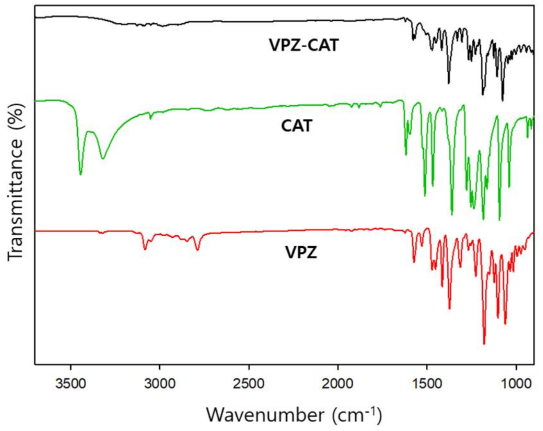

Figure S31. FT-IR spectra of VPZ, CAT, and VPZ-CAT prepared by RC

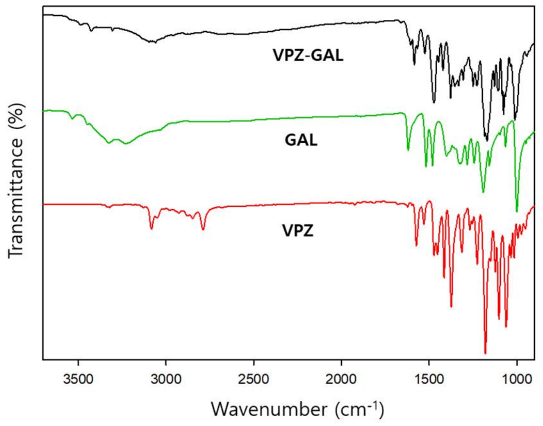

Figure S32. FT-IR spectra of VPZ, GAL, and VPZ-GAL prepared by RC.
